# Knowledge and Attitude of King Khalid University Students Toward Common Issues of Ear, Nose, and Throat

**DOI:** 10.7759/cureus.64720

**Published:** 2024-07-17

**Authors:** Ibrahim N Al Sulaiman, Wafaa S Taishan, Abdulaziz A Qibti, Nada A Asiri, Abdulkhaliq Abdullah A Oraydan, Yasser A Alyahya, Nouf K Alshehri, Osama A Asiri, Abdulrhman H Alzahrani

**Affiliations:** 1 Medicine, Najran University, Najran, SAU; 2 Medicine/Otolaryngology, Al-Baha University, Al-Baha, SAU; 3 Otolaryngyology, King Khalid University, Abha, SAU; 4 Medicine, King Khalid University, Abha, SAU; 5 Medicine and Surgery, King Khalid University, Abha, SAU; 6 General Surgery, King Fahad General Hospital, Jeddah, SAU

**Keywords:** saudi arabia, health education, university students, ent knowledge, otorhinolaryngology

## Abstract

Introduction

Ear, nose, and throat (ENT), often known as otorhinolaryngology (ORL), is a subspecialty within medicine that specializes in diagnosing and treating conditions affecting the head, neck, and ears. Understanding ORL is essential for treating common ENT issues, avoiding complications, and preserving quality of life. These diseases can affect numerous physiological processes, including taste, smell, speaking, breathing, swallowing, hearing, and secretion clearance. In order to guide interventions for improved ENT health, our study sought to evaluate university students' degree of ORL-related knowledge.

Methodology

It is a cross-sectional study conducted among students at King Khalid University in Saudi Arabia. Validated surveys are used to collect data via the Internet, including online and email-based data collecting. Data are cleaned in Excel and analyzed by IBM SPSS (IBM Corp., Armonk, NY).

Results

Our study included 131 Saudi university students in Abha City, revealing significant knowledge gaps and misconceptions regarding ENT issues. Despite high awareness of certain topics like flu vaccination (87.8%) and the association between hearing loss and social life (95.4%), misconceptions persisted, such as the belief in vitamin C's efficacy against influenza (51.9%). Notably, 47.3% displayed good knowledge, 26.7% showed moderate knowledge, and 26% demonstrated poor knowledge about ENT issues. Significant associations were found between knowledge levels and age (p<0.001), academic level (p<0.001), and previous surgery related to ENT issues (p=0.014).

Conclusion

Our study revealed that 47.3% of participants have good levels of knowledge regarding ENT problems, with significant associations found with age, academic level, previous ENT surgery, and diagnoses of throat issues. Education campaigns should target specific demographics to improve the overall understanding of ENT health.

## Introduction

Ear, nose, and throat (ENT), often known as otorhinolaryngology (ORL), is a subspecialty within medicine that diagnoses and treats disorders affecting the head and neck. It is impossible to exaggerate how important it is for the general public to be aware of ENT issues because many common ENT diseases substantially influence a person's quality of life and can worsen if left untreated [1,2]. ENT illnesses, however, are extremely important because of the morbidities that can lead to impairment of physiological function. Flavor, smell, speech, breathing, swallowing, lower respiratory tract protection, hearing, and secretion clearance are among the challenges described above [3,4]. ENT-related symptoms are one of the main causes of consultations with primary care physicians worldwide [5]. The World Health Organization estimates that 278 million people worldwide experience bilateral hearing loss that ranges from moderate to severe [6]. According to reports, 16.1% of adult US citizens experienced hearing loss between 2003 and 2004 [7]. An estimate from 2004 put the prevalence of rhinosinusitis among nasal illnesses in the adult US population at 16% [8]. To avoid issues and enhance the general health of those impacted, early detection and effective treatment of these disorders are crucial [9]. There was not enough information provided about local attitudes and knowledge of ENT-related issues. About 2.3% of residents of the city of Riyadh were found to have extraordinary knowledge, and 18.4% to have a high degree of knowledge, according to prior research. The majority of people (79.4%) know very little about conditions related to the ENTs [10]. In another survey, which involved college and high school students in Makkah City, a sizable percentage of participants (42.22%) showed a moderate degree of comprehension of ENT concerns, while 41.48% had a low level of understanding. However, only 16.30% of the participants demonstrated a high level of expertise in this field [11].

Many university students in Abha City, which is in the southern part of Saudi Arabia, may be at risk for ENT-related problems as a result of a variety of variables, including local lifestyle choices, environmental circumstances, and restricted access to specialist healthcare services [12]. Unfortunately, little is known regarding King Khalid University students' understanding of and familiarity with frequent ENT-related problems as of yet.

Accordingly, this cross-sectional study aims to address this knowledge gap and assess the level of knowledge and awareness of common ENT-related issues among university students in Abha City, Saudi Arabia. This study's findings will help identify gaps in ENT knowledge and awareness, inform the development of targeted educational interventions, and contribute to the improvement of ENT health outcomes in the region.

## Materials and methods

This was a cross-sectional study conducted from March 10 to May 10, 2024 to assess the level of knowledge and awareness of common ENT-related issues among university students in Abha City, Saudi Arabia, to determine and address the knowledge gap of common ENT-related issues. Students at King Khalid University, Saudi Arabia, and those aged between 18 and 30 years, who agreed to participate in the current survey, were included in the study.

The study was approved by the Institutional Research Board of King Khalid University (approval number: ECM#2024-1405). The participants were informed about the study aims and assured of data confidentiality, and consent was obtained from each participant before participating in the study.

Sample size

The sample size was calculated using Cochran’s equation with a precision level of ±5% and a confidence level of 95%. The estimated number of students is 200 and the calculated sample size was 131.

Data collection

An anonymous, self-administered validated electronic questionnaire was distributed through social media WhatsApp, and Telegram among students from different faculties which include Medicine, Applied Medical Sciences, Pharmacy, and the College of Engineering of King Khalid University, Saudi Arabia. The questionnaire was available in two languages, English and Arabic, and the participants were free to choose the language they preferred. The questionnaire collected demographic data, past medical or clinical history of any problem related to ENT and knowledge about problems related to ENT.

Statistical analysis plan

A comprehensive statistical analysis was conducted on the dataset, encompassing both descriptive and inferential methodologies. Firstly, a descriptive analysis is conducted to summarize the demographic characteristics of the participants, which include age, gender, and other features. This provides an overview of the study population. Subsequently, inferential analyses, such as the Chi-Square/Fisher Exact test, are employed to see the association between the awareness and knowledge level between different parameters. Statistical significance is established at a p-value of 0.05 or lower and a 95% confidence interval. All statistical analyses are executed using IBM's SPSS Software, version 29.0.0 (IBM Corp., Armonk, NY).

## Results

Our study surveyed 131 university students in Abha City, Saudi Arabia, with a mean age of 23.3 years (SD = 1.7), primarily aged between 18 and 24 years (n=90, 68.7%). All participants were Saudi nationals, and the majority (n=124, 94.7%) studied in health colleges, predominantly in the Faculty of Medicine and Surgery (n=108, 82.5%) (Table 1). Regarding educational background, 38.9% (n=51) had basic education (first to ninth grade), while 29.0% (n=38) were interns. In the event of sudden hearing loss, 93.1% (n=122) of participants would approach a hospital, while only 6.9% (n=9) would consult a general or ENT doctor.

**Table 1:** Sociodemographic and other parameters of all participants (n=131) (N) Frequency, (%) Percentage

Variable		N (%)
Age	18-24 years	90 (68.7)
	25-30 years	41 (31.3)
	Mean (SD)	23.3 (1.7)
	Range	20-28
Studying in health college	Yes	124 (94.7)
	No	7 (5.3)
Department of study	Faculty of Medicine and Surgery	108 (82.5)
	College of Applied Medical Sciences - Public Health	9 (6.9)
	Faculty of Pharmacy	7 (5.3)
	Other	7 (5.3)
Academic level	Basic education	51 (38.9)
	Secondary education	18 (13.7)
	University education	24 (18.3)
	Internship	38 (29.0)
Action/response of participants in case of sudden hearing loss	Consult General/ENT doctor	9 (6.9)
	Approach hospital	122 (93.1)

Table 2 shows the participants' past medical or clinical history, where 27.5% (n=36) reported previous diagnoses of ear problems, while 22.1% (n=29) had nasal issues, and 12.2% (n=16) experienced throat problems. Furthermore, 18.3% (n=24) disclosed a history of surgery related to the ENT. Conversely, the majority had no prior diagnoses of the ear (72.5%, n=95), nasal (77.9%, n=102), or throat (87.8%, n=115) issues. 

**Table 2:** Past medical or clinical history of participants (n=131) (N) Frequency, (%) Percentage

		N (%)
Have you previously been diagnosed with ear problems?	No	95 (72.5)
	Yes	36 (27.5)
Have you previously been diagnosed with nasal problems?	No	102 (77.9)
	Yes	29 (22.1)
Have you previously been diagnosed with throat problems?	No	115 (87.8)
	Yes	16 (12.2)
Have you ever had previous surgery related to the ear, nose or throat?	No	107 (81.7)
	Yes	24 (18.3)

Table 3 shows the assessment of knowledge about ENT issues among participants. A significant majority recognized that cotton swabs are a safe way to clean the ear (80.2%, n=105) and that antibacterial medications are not appropriate for treating upper respiratory tract infections (61.1%, n=80). Additionally, a considerable proportion understood the importance of annual flu vaccination as a preventative measure (87.8%, n=115), and that individuals with diabetes and high blood pressure should receive the seasonal influenza vaccine (84.0%, n=110). However, there were misconceptions regarding the effectiveness of vitamin C in treating and preventing influenza, with 51.9% (n=68) believing it to be true, while 34.4% (n=45) correctly believed it doesn’t help. Notably, 38.2% (n=50) correctly identified that vertigo is not another term for dizziness, while 95.4% (n=125) recognized that hearing loss may indeed affect an individual's social life. Moreover, a significant majority understood that continuous exposure to loud sounds can lead to hearing loss (91.6%, n=120) and that some infections in the inner and middle ear can cause vertigo (93.1%, n=122). Additionally, the majority recognized that not every earache necessarily indicates a middle ear infection (84.0%, n=110). Notably, 64.1% (n=84) recognized that tilting the head back is not the correct way to deal with a nosebleed, while 55.7% (n=73) understood that using nasal congestion relief drops for long-term use is not safe. Furthermore, a significant majority correctly identified that tonsillectomy does not cause obesity (28.2%, n=37) or a weakened immune system (31.3%, n=41). Additionally, the majority correctly understood that constant screaming can indeed cause vocal cord disorders (88.5%, n=116).

**Table 3:** Assessment of knowledge about problems related to ENT (n=131) (*) Correct Answers, (N) Frequency, (%) Percentage ENT - Ear, nose, and throat

Item		N (%)
Cotton swabs are a safe way to clean the ear	No	8 (6.1)
	Yes*	105 (80.2)
	Don't know	18 (13.7)
Antibacterial medications are not an appropriate treatment for upper respiratory tract infections?	No	33 (25.2)
	Yes*	80 (61.1)
	Don't know	18 (13.7)
It is recommended to get the flu vaccine annually as a preventative measure	No	7 (5.3)
	Yes*	115 (87.8)
	Don't know	9 (6.9)
It is recommended that people with diabetes and high blood pressure get the seasonal influenza vaccine	No	4 (3.1)
	Yes*	110 (84.0)
	Don't know	17 (13.0)
Vitamin C treats and prevents influenza	No*	45 (34.4)
	Yes	68 (51.9)
	Don't know	18 (13.7)
Vertigo is another term for dizziness	No*	50 (38.2)
	Yes	70 (53.4)
	Don't know	11 (8.4)
Hearing loss may affect an individual's social life	No	5 (3.8)
	Yes*	125 (95.4)
	Don't know	1 (0.8)
Continuous exposure to loud and annoying sounds may cause hearing loss	No	4 (3.1)
	Yes*	120 (91.6)
	Don't know	7 (5.3)
Some infections in the inner ear and middle ear cause vertigo	No	2 (1.5)
	Yes*	122 (93.1)
	Don't know	7 (5.3)
Not every earache is necessarily a middle ear infection	No	5 (3.8)
	Yes*	110 (84.0)
	Don't know	16 (12.2)
The correct way to deal with a nosebleed is to tilt the head back	No*	84 (64.1)
	Yes	21 (16.0)
	Don't know	26 (19.8)
Using nasal congestion relief drops is safe for long-term use	No*	73 (55.7)
	Yes	12 (9.2)
	Don't know	46 (35.1)
Tonsillectomy may cause obesity	No*	37 (28.2)
	Yes	23 (17.6)
	Don't know	71 (54.2)
Tonsillectomy may cause a weakened immune system	No*	41 (31.3)
	Yes	55 (42.0)
	Don't know	35 (26.7)
Constant screaming causes vocal cord disorders	No	3 (2.3)
	Yes*	116 (88.5)
	Don't know	12 (9.2)

**Figure 1:**
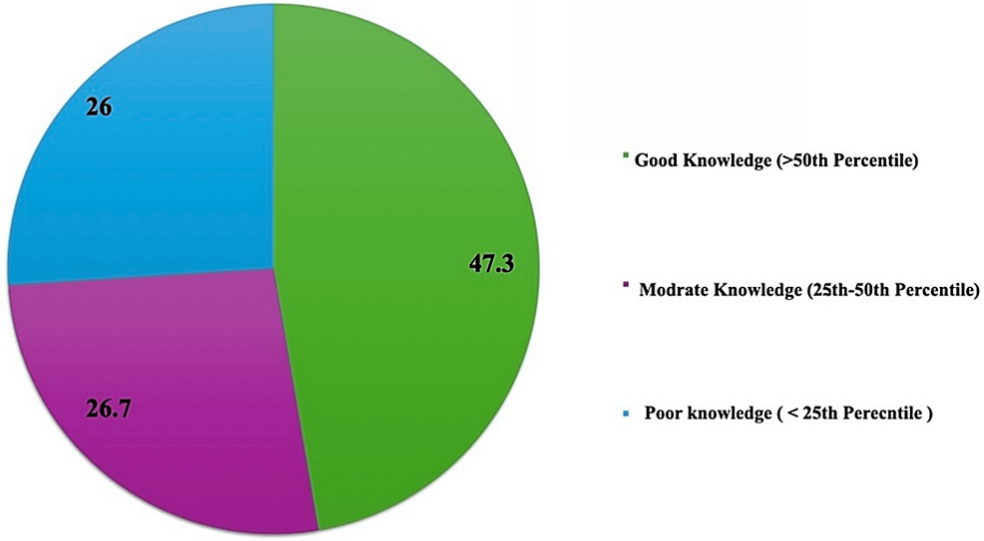
The knowledge score of participants (n=131)

The knowledge score in Figure 1 shows the distribution among participants. The pie chart demonstrates that 47.3% of participants exhibited good knowledge, scoring above the 50th percentile. Additionally, 26.7% displayed moderate knowledge, falling within the 25-50th percentile range. Conversely, 26% of participants demonstrated poor knowledge, scoring below the 25th percentile.

The association between knowledge levels about ENT problems and various parameters among the surveyed participants. Significant associations were observed between knowledge levels and age (p < 0.001), academic level (p < 0.001), previous surgery related to the ENT (p = 0.014), and previous diagnoses of throat problems (p = 0.031). Specifically, a higher proportion of participants were aged 25-30 years (97.6%) and exhibited moderate/high knowledge compared to those aged 18-24 years (63.3%) (Table 4). Similarly, participants with higher academic levels had moderate/high knowledge about ENT issues (92.1% with Internship compared to 56.9% with less than secondary education). Notably, those who underwent previous surgery related to ENT issues showed a lower proportion of moderate/high knowledge and a higher proportion of poor knowledge level (45.8% with low knowledge and 54.2% with moderate/high knowledge as compared to those without previous surgery). Similarly, participants diagnosed previously with throat problems also showed a lower proportion of moderate/high knowledge and a higher proportion of poor knowledge level (50.0% with low knowledge and 50.0% with moderate/high knowledge compared to those without previous throat problems). In contrast, no significant associations were found with parameters such as studying in a health college, department of study, approach in case of sudden hearing loss, or previous ear and nasal problems diagnoses.

**Table 4:** Association of knowledge level about problems related to ENT with different parameters (a)Chi-Square, (b) Fisher’ Exact Test ENT - Ear, nose, and throat

		Knowledge/awareness level about ENT problems		Sig. value
		Poor knowledge, N=34	Moderate/high knowledge, N=97	
		N (%)	N (%)	
Age	18-24 years	33 (36.7)	57 (63.3)	<0.001^a^
	25-30 years	1 (2.4)	40 (97.6)	
Studying to health college	No	4 (57.1)	3 (42.9)	0.074^ b^
	Yes	30 (24.2)	94 (75.8)	
Which department	Faculty of Medicine and surgery	25 (23.1)	83 (76.9)	0.136^ b^
	Applied Medical Sciences - Public Health	2 (22.2)	7 (77.8)	
	Faculty of Pharmacy	3 (42.9)	4 (57.1)	
	Other (Non-health related)	4 (57.1)	3 (41.9)	
Academic Level	< Secondary education	22 (43.1)	29 (56.9)	<0.001^b^
	Secondary education	6 (33.3)	12 (66.7)	
	University education	3 (12.5)	21 (87.5)	
	Internship	3 (7.9)	35 (92.1)	
In the event of sudden hearing loss or ear pain, what do you think is the appropriate course of action?	Consult General/ENT doctor	2 (22.2)	7 (77.8)	1.000^b^
	Approach hospital	32 (26.2)	90 (73.8)	
Previously diagnosed with ear problems?	No	26 (27.4)	69 (72.6)	0.549^a^
	Yes	8 (22.2)	28 (77.8)	
Previously diagnosed with nasal problems?	No	29 (28.4)	73 (72.6)	0.337^a^
	Yes	5 (22.2)	24 (82.8)	
Previously diagnosed with throat problems?	No	26 (22.6)	89 (77.4)	0.031^b^
	Yes	8 (50.0)	8 (50.0)	
Previously surgery related to ENT?	No	23 (21.5)	84 (78.5)	0.014^a^
	Yes	11 (45.8)	13 (54.2)	

## Discussion

ORL, or ENT, is crucial for diagnosing and treating disorders in these areas and the head/neck. ENT knowledge is vital as ENT conditions impact the quality of life and can lead to severe life-threatening complications. Lukama et al. showed that delayed diagnosis of benign ENT conditions may result in life-threatening complications (airway obstruction in laryngeal papilloma, intracranial sepsis in acute otitis media, and rhinosinusitis) [13]. Impairments include issues with taste, smell, speech, breathing, hearing, and secretion clearance [14]. Our study aimed to assess the knowledge and awareness of common ENT issues among King Khalid University students in Abha City, Saudi Arabia. Our findings revealed various levels of understanding among the participants, which we will now discuss in comparison with previous medical literature.

Notably, our study found a significant association between knowledge levels and age, academic level, and previous surgery related to ENT issues. Specifically, older participants and those with higher academic levels demonstrated a higher likelihood of moderate/high knowledge. This aligns with previous research indicating that older individuals and those with higher education levels tend to have better health-related knowledge due to increased exposure to health information and education opportunities. Jalaladdin et al. showed that female participants with bachelor's or university degrees and participants aged 20 and older showed superior knowledge [15]. Moreover, Assiri et al. showed that the participants older than 20 years had better knowledge of common ENT problems than those younger than 20 years showing the impact of age on knowledge level [16]. Additionally, the association between previous surgery related to ENT issues and lower knowledge levels suggests that uneducated or less educated patients experience more ENT problems and face surgeries to manage these problems may be due to poor understanding and awareness about ENT issues. However previous study by Jalaladdin et al. showed that past ENT medical and surgical histories did not show a statistically significant association with their knowledge of ENT issues [15].

Moreover, our study revealed varying levels of awareness regarding common ENT issues among participants. While a significant proportion demonstrated good knowledge of certain topics, such as the importance of annual flu vaccination and the association between hearing loss and social life, misconceptions or knowledge gaps were also evident, particularly regarding the effectiveness of vitamin C in preventing influenza and the correct management of nosebleeds. Gorton et al. showed that high doses of vitamin C relieved and prevented cold and flu symptoms compared to controls [17]. Tr et al. showed that vitamin C has an important role in reducing the risk of nose bleeding. Vitamin C is essential for strengthening blood vessels [18].

Moreover, our study found that a higher proportion of participants with moderate/high knowledge levels would approach a hospital in the event of sudden hearing loss, compared to those with poor knowledge levels. This finding is consistent with those of Alshehri et al., which reported a substantial level of knowledge about the appropriate action to be taken in case of a medical emergency regarding ORL-related issues [19]. This suggests that individuals with better knowledge of ENT issues may be more likely to seek appropriate medical care in response to symptoms. Similarly, Badane et al. showed that TB patients showed good knowledge of TB signs, transmission, and thus healthcare-seeking behavior [20]. However, no significant associations were found between knowledge levels and previous diagnoses of ear and nasal problems, indicating that knowledge alone may not always translate into health-seeking behavior. Whereas a previous study by Alkholaiwi et al. showed various significant associations between knowledge and demographic variables. Age (p<0.001), gender (p<0.001), marital status (p<0.001), and education level (p=0.001) were all correlated with knowledge. Additionally, employment status was linked to knowledge (p=0.007). Binary logistic regression showed older participants (41-60 years) were more likely to have good ENT knowledge. Males had lower odds of acceptable knowledge than females. Singles had lower odds than married individuals [21]. There are several limitations of our study, which include potential recall bias due to self-reported data, limiting the accuracy of responses. The study's cross-sectional design restricts establishing causality between variables. The sample's homogeneity, consisting primarily of university students in Abha City, Saudi Arabia, may limit the generalizability of findings to broader populations.

## Conclusions

Our study provides valuable insights into the knowledge and awareness of ENT issues among King Khalid University students in Abha City, Saudi Arabia. While certain demographic factors, such as age, education level, and personal experiences with ENT problems, are associated with better knowledge levels, there are still areas of misconceptions and gaps in awareness that need to be addressed. Targeted educational interventions and healthcare provider efforts are essential for improving ENT-related knowledge and promoting appropriate health-seeking behavior among this population.
